# Social cognition training improves recognition of distinct facial emotions and decreases misattribution errors in healthy individuals

**DOI:** 10.3389/fpsyt.2022.1026418

**Published:** 2022-11-04

**Authors:** Samantha Evy Schoeneman Patel, Kristen M. Haut, Erin Guty, David Dodell-Feder, Abhishek Saxena, Mor Nahum, Christine I. Hooker

**Affiliations:** ^1^Department of Psychiatry and Behavioral Sciences, Northwestern University Feinberg School of Medicine, Chicago, IL, United States; ^2^Department of Psychiatry and Behavioral Sciences, Rush University Medical Center, Chicago, IL, United States; ^3^Department of Psychology, Pennsylvania State University, State College, PA, United States; ^4^The Charleston Consortium Psychology Internship, Ralph H. Johnson Veterans Affairs Medical Center, Medical University of South Carolina, Charleston, SC, United States; ^5^Department of Psychology, University of Rochester, Rochester, NY, United States; ^6^Department of Neuroscience, University of Rochester Medical Center, Rochester, NY, United States; ^7^School of Occupational Therapy, Faculty of Medicine, Hebrew University, Jerusalem, Israel

**Keywords:** social cognition, cognitive remediation, neuronal plasticity (MeSH), facial emotion recognition (FER), schizophrenia, Parkinson’s disease

## Abstract

Facial emotion recognition is a key component of social cognition. Impaired facial emotion recognition is tied to poor psychological wellbeing and deficient social functioning. While previous research has demonstrated the potential for social cognition training to improve overall facial emotion recognition, questions remain regarding what aspects of emotion recognition improve. We report results from a randomized controlled trial that evaluates whether computerized social cognition training can improve recognition of distinct facial emotions in healthy participants. This investigation was designed to better understand the therapeutic potential of social cognition training for individuals with neuropsychiatric disorders. Fifty-five healthy adult participants were randomly assigned to an internet-based intervention during which they either completed social cognition training (SCT) or played control computer games (CON) for 10.5 h over 2–3 weeks. Facial emotion recognition was measured with the Penn ER-40, which was conducted before and after training. The following variables were collected and analyzed: facial emotion recognition accuracy for each emotion (i.e., anger, fear, happy, neutral (no emotional expression), and sad), reaction times for each emotion, and response error types (i.e., frequency of an emotion being chosen incorrectly, frequency of an emotion being missed, and frequency of an emotion being confused for another particular emotion). ANOVAs and *t*-tests were used to elucidate intervention effects both within and between groups. Results showed that the SCT group improved their accuracy for angry and neutral faces. They also improved their reaction times for neutral, fearful, and sad faces. Compared to the CON group, the SCT group had significantly faster reaction times to neutral faces after training. Lastly, the SCT group decreased their tendency to confuse angry faces for no emotional expression and to confuse no emotional expression for sad faces. In contrast, the CON group did not significantly improve their accuracy or reaction times on any emotional expression, and they did not improve their response error types. We conclude that social cognition training can improve recognition of distinct emotions in healthy participants and decrease response error patterns, suggesting it has the potential to improve impaired emotion recognition and social functioning in individuals with facial emotion recognition deficits.

## Introduction

Facial emotion recognition is a major component of social cognition, that, when impaired, leads to compromised social functioning (1–8) and poor psychological wellbeing (9). These long-term consequences are, most likely, the cumulative effect of abnormally slow or erroneous facial recognition judgments during many, successive interpersonal interactions over time. For example, individuals who regularly misperceive others as angry might, in turn, act coldly toward others and alienate them, leading to interpersonal rejection, social isolation, and difficulties in work or school settings. Over the course of many years, these social problems can get progressively worse, ultimately leading to unemployment and/or homelessness (4). This relationship between emotion recognition ability and social outcomes underscores the importance of identifying and treating emotion recognition deficits. Indeed, a large body of research demonstrates that facial emotion recognition deficits are common across populations with neuropsychiatric disorders as well as those who are at risk for a neuropsychiatric illness (e.g., due to genetics or emerging symptoms) but do not meet full diagnostic criteria for a neuropsychiatric disorder. Interestingly, these emotion recognition deficits manifest differently in different people and populations. Individuals with Parkinson’s disease have trouble recognizing anger and fear (10–12). Individuals with major depressive disorder often mistake neutral faces for sad faces, (13, 14), and this tendency itself has been to found perpetuate depressive symptoms (15, 16). Individuals with schizophrenia have trouble recognizing neutral, angry, and fearful faces and often misperceive other emotions as anger (4, 17–21). Relatives of individuals with schizophrenia, who are considered neuropsychiatrically healthy, have trouble with emotionless faces, to which they misattribute negative emotions (22).

Importantly, facial emotion recognition skills can be measured with behavioral assessments, and, thus, deficits can be identified. Moreover, there is extensive research demonstrating that facial emotion recognition performance is supported by a well-defined neural network, and deficits, in both healthy and neuropsychiatric populations, are associated with aberrations in this neural system (23–27). Building on these findings, researchers have proposed that standardized neurocognitive batteries should include behavioral assessments of emotion recognition as identifying deficits can indicate potential neural dysfunction as well as potential vulnerabilities in short-term and long-term social functioning (8).

Therefore, given that deficient facial emotion recognition is common and significantly impacts social functioning, interventions improving facial emotion recognition should be a priority. Furthermore, facial emotion recognition is an optimal intervention target because the underlying neural network is well-defined and because there are validated assessments that measure facial emotion recognition performance. However, limited interventions exist that successfully strengthen facial emotion recognition. Pharmacological treatments do not consistently improve facial emotion recognition and other social cognition deficits (28, 29). Group-based social skills training has been shown to improve overall facial emotion recognition in individuals with schizophrenia (30), bipolar disorder (31), autism (32), and Parkinson’s disease (33), but they often take a one-size-fits-all approach when, as discussed above, individuals may have different deficits requiring personalized, adapted programs (34). Furthermore, many individuals do not have access to mental health clinics or group therapy environments, and access has been even more limited in the context of the COVID-19 pandemic. Lastly, these training programs were not developed to explicitly target the neural network underlying facial emotion recognition, which, as stated above, is often impaired in individuals with facial emotion recognition deficits; thus, these existing programs may not address the root cause of the problem (35).

Another problem is that existing interventions are not designed to address individual differences in emotion recognition performance nor does the evaluation of intervention efficacy consider success for individual performance patterns. As stated above, different individuals struggle recognizing different facial emotions, warranting a type of intervention that can address a variety of facial emotion recognition deficits. Therefore, evaluations of facial emotion recognition interventions should investigate how interventions impact recognition of distinct emotions. However, little work has been done, to our knowledge, looking at whether and/or how facial emotion recognition interventions impact recognition of distinct emotions. With perhaps one exception (36), most investigations of facial emotion recognition interventions do not differentiate between recognition of different emotions and instead monitor performance changes in overall emotion recognition accuracy and reaction time.

SocialVille (Posit Science, San Francisco, CA, USA) is an online social cognition training program that, unlike many other social cognition interventions, utilizes the principles of neuroplasticity to improve social functioning. These principles dictate that training should *engage and strengthens the specific neural networks of interest* while being *adaptive, intensive, and reinforcing* in order to promote desired behavioral changes (37). Therefore, in SocialVille trainees practice both lower-level social cognition skills, including facial emotion recognition, and higher-level social cognition skills, such as theory of mind, across 12 exercises known to specifically *engage the neural networks* underlying social cognition (35, 38, 39). To ensure the program is *intensiv*e, *adaptive*, and continues to consistently *engage* the brain regions of interest, the program becomes progressively more difficult as participants improve their performance. Finally, it is *reinforcing* because it rewards correct responses. Preliminary imaging evidence has shown that neuroplasticity-based programs comparable to SocialVille successfully engage the neural network underlying facial emotion recognition and improve facial emotion recognition (40, 41). Overall, these programs have been shown to improve social cognition in individuals with schizophrenia (35, 38, 42, 43), those at high risk for psychosis (44), and healthy adults (45). Furthermore, SocialVille can improve access to those who may be unable to receive in-person interventions: the exercises are browser-based and can be performed remotely on any internet-connected computer.

In this pilot randomized controlled trial, conducted in healthy participants, we evaluated whether social cognition training, using the SocialVille program, fosters prompt and accurate recognition of distinct emotions. We used a double-blind, modified block-randomized design with two groups: (1) the experimental group who completed social cognition training (SCT group) and (2) an active control group who played computer games (CON) that did not contain social content. We hypothesized that, because SocialVille is designed to target the brain regions responsible for higher- and lower-level social cognition skills, including facial emotion recognition, and because it utilizes the principles of neuroplasticity, it will: (1) improve participants’ accuracy in recognizing distinct emotions; (2) improve participants’ speed in recognizing distinct emotions and (3) decrease response error patterns (i.e., repeatedly mistaking one facial emotion for another emotion).

## Materials and methods

### Participants

The present study focuses on one assessment that was conducted along other assessments in a pilot randomized controlled trial, conducted in healthy participants, that examined the effects of social cognition training (45). The goal of this pilot study was to identify key intervention targets for a subsequent study that investigated the impact of social cognition training on individuals at risk for psychosis (35).

This study was approved by the Harvard Institutional Review Board (IRB Protocol # 14-3110). All participants received a description of the study before they provided written informed consent. The recruitment procedures have been previously described (45). In short, individuals between ages 18 and 30 years of age were recruited from the local community (greater metro area of Boston, MA). Individuals were excluded for: (1) an Axis I Disorder or substance use disorder in the last 5 months per the Structured Clinical Interview (SCID) (46); (2) history of a major medical illness, neurological problem, or loss of consciousness > 30 min due to head trauma; (3) an IQ < 70 per the North American Adult Reading Test (NAART) (47) or a reading disability preventing them from completing the study; and/or (4) a contraindication to MRI.

### Study protocol

At baseline, study staff collected demographics information, including Gender, Age, and Years of education. Participants completed the NAART to assess their IQ. Participants completed neuroimaging and a battery of behavioral assessments before and after training. Participants were assigned either to the social cognition training group (SCT), which was the intervention group, or a computer game group, which was the active control group (CON). Using a block randomization procedure, the first 20 participants were assigned to the SCT group, and the second 20 participants were assigned to the CON group, after which subjects were alternately assigned to the SCT group or the CON group in pairs to account for age and gender. Group assignments were double blind: the experimenters who tested the participants were blind to the participants’ assignments; the participants themselves were only told they would be doing “brain training games” but were unaware of the study hypotheses or whether they were in the intervention group.

### Study interventions

The SCT and CON interventions have been previously described (45). As stated above, this SCT intervention utilized the principles of neuroplasticity by targeting the neural networks of interest in order to promote improved social behaviors and ultimately improved social function. It consisted of 12 exercises that are known to *engage the neural networks* underlying social cognition; these exercises focused on improving facial emotion recognition, perspective taking, navigation of social interactions, and theory of mind (Table 1). The exercises were *adaptive* and became more or less difficult to ensure participants were continuously challenged and thus *engaged*. Participants received badges within the program when they answered correctly to ensure the program was *reinforcing.* Participants also received feedback after each trial and could obtain feedback regarding their progress throughout the intervention as a whole. Participants in the CON group performed computer games without any social content, such as solitaire and word searches (Table 2).

**TABLE 1:** List of exercises in the social cognition training (SCT) intervention.

SCT exercises	
Exercise name	Description
Name that feeling	Select the label which correctly describes the facial expression of the rapidly presented target face (still image).
Emotion motion	Select the label which correctly describes the facial expression of the target face (video clip).
Voice choice	Select the label which correctly describes the target vocal affect (voice prosody).
Second that emotion	A memory game for facial expression: match pairs of cards that express the same facial affect.
Second that intonation	A memory game for vocal expression: match pairs of cards that express the same vocal affect (prosody).
Match that feeling	Select the face whose expression matches that of the rapidly presented target face.
Face it: flashback	Correctly memorize an increasingly longer sequence of faces.
Recognition	Select the target face from an array of faces presented from various angles.
Face facts	Memorize visually presented social facts about individuals presented serially.
Say what?	Decide how would a person respond in a given situation (audio scene).
What happened?	Decide what most likely happened given the reaction in the clip.
Social scenes	Rate the likelihood of people’s reactions and feelings in certain social situations.

**TABLE 2:** List of activities in the control (CON) intervention.

CON activities	
Activity name	Description
Chinese checkers	Move your pieces to the opponent’s end by moving or jumping over pieces.
Sudoku	Fill each square into the puzzle with a number (1–9) given rules.
Reversi	Try to have the majority of the disks in the game present your color.
Double klondike solitaire	Player must stack cards alternating in color in descending order with the goal of completing A-K stacks of the same suit.
Tri peaks solitaire	The object of the game is to remove all the cards that make up the “three peaks.” Player must stack the cards present on the “peaks” to the card on the bottom.
Brick breaking hex	Click on a group of blocks with the same color. To remove individual blocks, you lose one of your stars. The goal is to get rid of all the blocks before your stars.
Brick squasher II	Use the mouse to control the board to bounce the balls and destroy the bricks. Some bricks require a few hits, and some bricks are indestructible.
Gem swap	Swap adjacent gems to create 3 or more in a row to remove the gems.
War ship	Hide your ship, then press OK. Take turns with the computer player to search for the opponent’s hidden ship. The object of the game is to find your opponent’s ships and sink them before they find and sinks yours.
A maze race	There are two balls: the green one is designated to the participant, and the red one is the computer player. The participant must find the “Flag” or end point before the computer does.
Lineup 4	Participant and computer player take turns dropping colored disks from the top into a grid. The participant must connect four yellow disks in a row (vertically, horizontally, or diagonally) before the opponent.
Word search II	Letters are placed in a grid, and the participant must find the specified list of words hidden within the grid.
Crossword puzzle	The participant must hover over the line of blocks to view the hint.

Both the SCT and CON interventions were accessed *via* the same website and could be performed on any computer at the participants’ convenience. All participants, regardless of group, were asked to complete a total of 15 sessions. Each session lasted approximately 45 min; therefore, all participants received 10.5 h of training. Training was self-directed, so participants were able to complete 1–3 training sessions a day for 5 days a week. Overall, training lasted 2–3 weeks depending on how quickly participants completed their sessions. Training was monitored remotely by research staff who would reach out to participants who had missed 2 days of training to remind them to resume their training. Participants who did not resume training after these reminders and had missed more than 2 days of training were regarded as non-adherent and excluded.

### Assessments

As stated above, all participants underwent a battery of assessments. A previous study by Haut et al. reported on a distinct domain of social cognition in this same sample of participants: empathic accuracy (45). This present study is concerned strictly with facial emotion recognition and misattribution errors as measured by the Penn Emotion Recognition Task (ER-40) – a computerized facial emotion recognition task that we selected for its high test-retest reliability and high construct validity (8). In this task, participants are instructed to identify the emotion displayed by a color picture of a face by using a mouse to click on of the following options listed next to the face: happy, angry, sad, fear, or no emotion (neutral). The participants begin with one practice trial after which feedback about accuracy is given. If needed, participants repeat the practice trial until they choose the correct response. After completing the practice trial, they move onto the actual assessment, which consists of 40 trials (i.e., 8 trials for each of the 5 emotions) shown in a randomized fashion. There are 4 female faces and 4 male faces for each emotion. For each emotion, there are 4 high-intensity (extreme) and 4 low-intensity (subtle) expressions. Emotion type, emotion intensity, age, gender, and ethnicity of the faces are balanced across trials (8, 48–50). The participants’ level of arousal in response to each picture was not measured. Participants completed the task before and after the intervention; the following behavioral variables were collected for each trial: response choice, whether a trial was correct or incorrect, and reaction time (RT) (amount of time between stimulus presentation and mouse click response) in milliseconds (ms).

### Statistical analyses

All analyses were performed with either Microsoft Excel for Mac Version 16.52 (Microsoft, Redmond, WA) or SPSS Version 27 (IBM, Armonk, NY).

Data for each participant were inspected for outliers and other abnormalities. An outlier was defined as any trial with an RT 3 standard deviations above or below the mean of all reaction times over both groups and time points. 0.6% (25/4400) of trials had an RT 3 standard deviations above the mean and were removed from all analyses. No trial had an RT 3 standard deviations below the mean. All incorrect trials were included in accuracy analyses. RT analyses only included correct trials with the rationale that faster recognition only indicates improvement if said recognition is accurate. After removing outliers, we further removed 13% (580/4375) of the remaining trials because they were incorrect. Thus, a total of 3,795 trials were included in the RT analyses.

Five performance variables were analyzed for each emotion at each time point (i.e., pre- and post- intervention): accuracy, RT, miss frequency (MISS), False Positive, and specific misattribution errors. Accuracy for each emotion was calculated as the number of correct trials divided by the number of included trials. RT for each emotion was calculated as the average of the reaction times when an emotion was correctly recognized. MISS for each emotion was calculated as the number of times an emotion was missed divided by the total included trials (e.g., rate at which anger was not chosen when it was actually the correct choice). False Positive for each emotion was calculated as the number of times an emotion was incorrectly chosen divided by the total included trials (e.g., how often anger was chosen when it was the incorrect choice). Misattribution errors were calculated as the count of occurrences when a certain emotion was mistaken for another particular emotion (e.g., how often a neutral face was mistaken for an angry face).

Pearson chi-square tests and independent two-tailed sample *t*-tests were conducted to investigate potential differences in gender and age between those who completed the study, those who started the study but did not complete the study, and those who were excluded from the study. Pearson chi-square tests and independent two-tailed sample *t*-tests were also conducted to evaluate for any baseline differences between the SCT and CON groups in terms of Gender, Age, Years of education, estimated IQ, and ER-40 accuracy and RT for each emotion.

To test Hypotheses 1 (SCT will improve participants’ accuracy in recognizing distinct emotions) and 2 (SCT will improve participants’ speed in recognizing distinct emotions), repeated measures ANOVAs were conducted on accuracy and RT, respectively, with Group (SCT and CON) as the between-subjects variable and Time (Pre-intervention and Post-intervention) and Emotion as the within-subjects variables. ANCOVAs were then run with those same between-subjects and within-subjects variables while controlling for Gender, Age, Years of education, and IQ. Assumption checks for the ANOVAs were performed as follows for both the accuracy and RT datasets: we generated histograms for each of the datasets and confirmed the datasets were normally distributed; we used Levene’s test and confirmed homogeneity of variance; using Mauchly’s Test of Sphericity, we found that the assumption of sphericity was not met for Emotion and Emotion x Time (*p* < 0.001 for both) for both the accuracy and RT datasets; therefore, we used the multivariate test results to determine intervention effects, which are reported below. Assumption checks for the ANCOVAs were performed as follows for both the accuracy and RT datasets: we confirmed the assumption of homogeneity among the b-coefficients of the covariates by finding no significant (*p* > 0.05) between-subjects interaction effects between each covariate and Group; we confirmed the assumption of linearity between the covariates and the dependent variables by using a scatter plot to plot change in accuracy for each Group against each of the covariates and change in RT in each Group against each of the covariates: all relationships between the covariates and dependent variables were linear.

All ANOVA and ANCOVA results are reported with Bonferroni corrections. Independent two-tailed sample *t*-tests and paired two-tailed sample *t*-tests were then conducted to elucidate any effects found in the ANOVA analyses for between-subjects analyses and within-subjects analyses, respectively.

To test Hypothesis 3 (SCT will decrease participants’ patterns of response errors), we ran paired *t*-tests within each group comparing performance pre- and post-intervention for: (1) MISS frequencies for each emotion, (2) False Positive frequencies for each emotion, and (3) misattribution errors (i.e., how often a particular emotion was chosen instead of another specific emotion). *P* < 0.05 (Bonferroni corrected) was regarded as statistically significant for all analyses.

## Results

### Participants

Eighty-one individuals were screened, and 9 were excluded based on the above exclusion criteria. Ten participants then withdrew before being randomized, and 6 were either non-compliant with the training, withdrew, or were lost to contact before completing the study. Data were missing for one participant in the CON group. There were no significant differences in demographic characteristics between participants who were excluded and participants who remained in the study. Gender, χ^2^(1, *N* = 81) = 0.1.41, *p* = 0.70) was comparable between those who were excluded from the study, those who were randomized to the study but did not complete it/had missing data, and those who completed the study. Age was comparable between those who were excluded from the study and those who completed the study (*t*(72) = −0.16, *p* = 0.87). Age was also comparable between those who were randomized to the SCT group but did not complete the study and those who were randomized to the SCT group but did complete the study (*t*(33) = 0.531, *p* = 0.60). Given the CON group only had 1 participant with missing data, we did not run statistics comparing that 1 participant with the rest of the CON group who had completed the study.

The final sample consisted of a total of 55 subjects: 28 in the intervention group and 27 in the active control group. All further analyses concern this sample. Results from between-group comparisons showed no significant differences between the SCT and CON groups in terms of demographics (Table 3).

**TABLE 3:** Results from between-subjects comparisons for both gender (shown as number of men; number of women) and numerical demographic variables (shown as mean ± standard deviation).

	SCT (*N* = 27)	CON (*N* = 28)	χ ^2^	*p*
Gender	15;12	13;15	0.162	*p* = 0.688

**Numerical demographic variables**				

	**SCT (*N* = 27)**	**CON (*N* = 28)**	**t (df)**	***p***

Age (years)	24.93 ± 2.80	24.37 ± 3.22	0.68 (53)	0.50
Education (years)	16.36 ± 1.85	15.70 ± 1.96	1.27 (53)	0.21
NAART (estimated IQ)	120.75 ± 6.17	120.67 ± 5.99	0.05 (53)	0.96

### Baseline performance

The SCT and CON groups did not differ in their performance on the ER-40 task at baseline both in terms of accuracy for each emotion and in terms of RT for each emotion. Accuracy was highest for happy faces (>96%) and lowest for angry faces for both groups (Table 4).

**TABLE 4:** Results of ANOVAs evaluating for the effects of Group, Time, Emotion, and their interactions on accuracy and reaction time.

Accuracy					

Variable	df	F	Wilk’s Λ	Partial η2	*p*
Group	1.53	1.60	n/a	0.029	0.21
Time	1.53	4.16	0.93	0.07	0.05*
Emotion	4.50	92.70	0.11	0.88	< 0.01*
Group×Time	1.53	0.11	0.10	0.002	0.74
Group×Emotion	4.50	0.57	0.96	0.043	0.69
Time×Emotion	4.50	2.63	0.83	0.17	0.05*
Group×Time×Emotion	4.50	1.34	0.90	0.10	0.27

**Reaction time**					

**Variable**	**df**	**F**	**Wilk’s Λ**	**Partial η2**	***p***

Group	1.53	0.020	n/a	0.00	0.89
Time	1.53	0.77	16.11	0.23	< 0.01*
Emotion	4.50	33.81	0.27	0.73	< 0.01*
Group×Time	1.53	4.35	0.92	0.08	0.04*
Group×Emotion	4.50	1.16	0.92	0.085	0.34
Time×Emotion	4.50	2.13	0.86	0.15	0.091
Group×Time×Emotion	4.50	1.31	0.91	0.095	0.28

*Indicates a significant effect.

### Intervention effects

#### Multivariate analyses for accuracy

Results showed that Time, Emotion, and Time×Emotion had significant effects on accuracy (Table 4). When we ran an ANCOVA adjusting for Gender, Age, Years of education, and IQ, only Emotion remained significant (*F*(4,46) = 2.94, *p* = 0.03, Wilk’s Λ = 0.80, partial η2 = 0.20).

#### *Post-hoc* analyses for accuracy

**FIGURE 1:**
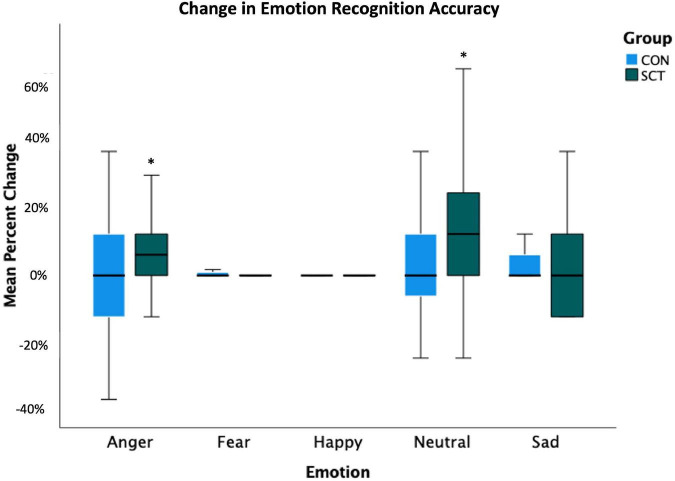
Change in emotion recognition accuracy (calculated as percent correct) following the intervention is shown. Pre-intervention accuracy percentages were subtracted from post-intervention accuracy percentages to yield the percent change values. *Indicates significant change from pre- to post-intervention (*p* < 0.05).

Although we did not find a significant Group×Time interaction effect, we did find effects of Time, Emotion, and Time x Emotion; therefore, we ran paired *t*-tests within each group to look at pre- to post-intervention changes in accuracy for each emotion. The SCT group improved significantly in their accuracy of recognizing angry (*t*(27) = −2.63, *p* = 0.01) and neutral (*t*(27) = −2.14, *p* = 0.04) faces (Figure 1 and Table 5).

**TABLE 5:** Pre- and post-training accuracy (% correct) and Reaction Time (ms) for each group.

Group	SCT (*N* = 27)		CON (*N* = 28)	
		
Time point	Pre-intervention	Post-intervention	Pre-intervention	Post-intervention

Accuracy (%)				
Anger	66 (13)	74 (12)*	66 (16)	68 (14)
Fear	95 (8)	94 (11)	94 (10)	95 (8)
Happy	99 (4)	96 (11)	96 (10)	97 (8)
Neutral	79 (24)	89 (16)*	80 (20)	84 (19)
Sad	92 (1)	93 (12)	88 (17)	89 (14)

**Reaction time (ms)**				

Anger	2340.80 (719.93)	2029.94 (734.71)	2181.88 (649.60)	2005.57 (524.23)
Fear	1993.38 (670.00)	1664.35 (321.06)*	2020.83 (90.90)	1847.35 (564.60)
Happy	1603.92 (472.94)	1498.25 (435.70)	1581.09 (419.88)	1538.47 (442.52)
Neutral	2418.39 (826.26)	1959.43 (417.31)*#	2247.21 (631.68)	2203.47 (671.58)
Sad	2077.68 (599.75)	1780.66 (48.14)*	1818.05 (411.04)	1779.59 (607.53)

Data shown as: Mean (SD). *Indicates *post-hoc t*-tests showed a significant change from pre- to post-intervention (*p* < 0.05). ^#^Indicates *post-hoc t*-tests showed a significant difference between groups at a particular time point (*p* < 0.05).

The CON group did not improve their accuracy significantly in the identification of any emotion.

Independent samples *t*-tests comparing post-intervention accuracy for each emotion between both groups yielded no significant differences.

#### Multivariate analyses for reaction time

**FIGURE 2:**
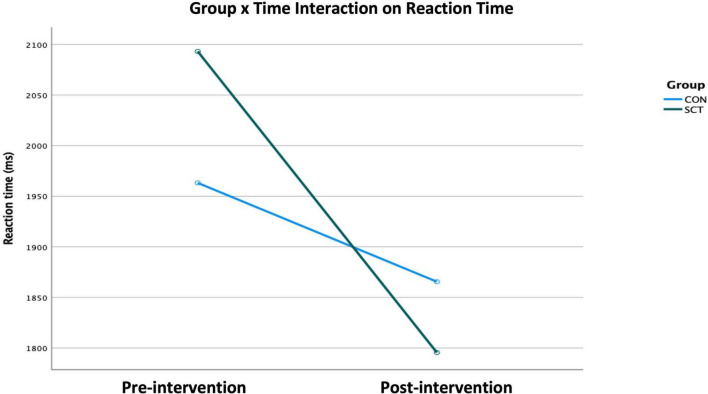
Effect of the Group×Time interaction on reaction time as generated × the ANCOVA examining the variables Group, Time, and Emotion while adjusting for Gender, Age, IQ, and Years of education (*p* < 0.05).

Time, Emotion, and Group×Time had significant effects on RT (Table 4). When we ran an ANCOVA adjusting for Gender, Age, Years of education, and IQ, the interaction of Group×Time remained significant (*F*(1,49) = 4.40, *p* = 0.04, Wilk’s Λ = 0.92, partial η2 = 0.02) (Figure 2).

#### *Post-hoc* analyses for reaction time

To further examine the significant Group×Time interaction on RT, we conducted paired *t*-tests within each group to look at pre- to post-intervention changes for each emotion type. In the SCT group, RTs for neutral (*t*(27) = 3.95, *p* < 0.001), fearful (*t*(27) = 2.72, *p* = 0.01) and sad (*t*(27) = 2.51, *p* = 0.02) faces significantly decreased, indicating significant improvement in RT. The RT for angry faces marginally decreased (*t*(27) = 1.90, *p* = 0.07).

**FIGURE 3:**
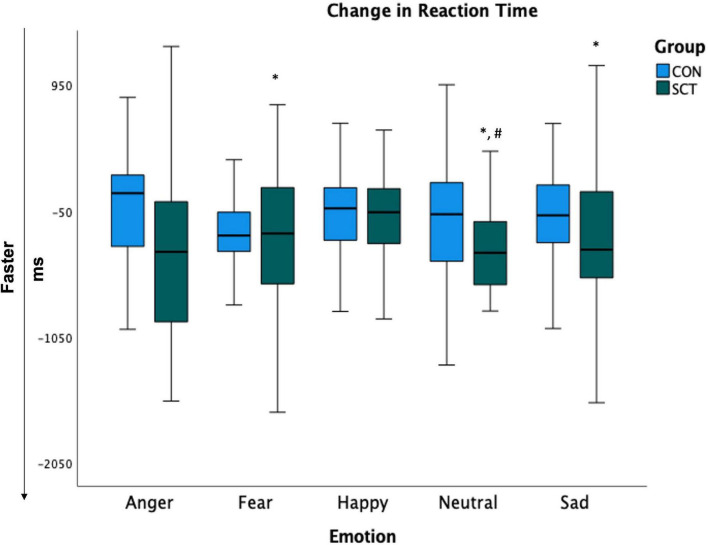
Change in reaction times (post-intervention minus pre-intervention). Larger negative values indicate greater improvement. *Indicates significant change from pre- to post-intervention (*p* < 0.05). ^#^Indicates significant difference between groups.

In the CON group, RTs for individual emotions did not significantly change (Figure 3).

Independent samples *t*-tests comparing SCT and CON groups for each emotion post-intervention showed that the RTs for neutral were significantly faster in the SCT group compared to the CON group (*t*(53) = −1.63, *p* = 0.032); RTs for fearful faces were marginally significantly faster in the SCT group than in the CON group (*t*(53) = −1.48, *p* = 0.09) (Table 5).

#### Analyses of error types

In the SCT group, anger MISS frequency (*t*(27) = 2.4, *p* = 0.02) and neutral MISS frequency (*t*(27) = 2.116, *p* = 0.04) significantly decreased from pre- to post-intervention. There were no significant changes in False Positive frequencies. The occurrence of SCT participants mistaking angry faces for no emotion (neutral) significantly decreased (*t*(27) = 2.5, *p* = 0.02), and the occurrence of SCT participants mistaking a neutral face for a sad face significantly decreased (*t*(27) = 2.3, *p* = 0.03).

In the CON group, there were no significant changes pre- to post-intervention in MISS frequencies, False Positive frequencies, and misattribution errors for any of the emotions.

## Discussion

This double-blind, randomized intervention trial with an active control group was conducted in healthy participants partly as a first step in developing and testing a social cognition intervention for use in individuals with neuropsychiatric disorders. An aim of this trial was to understand how social cognition training impacts healthy participants so that, when conducting comparable analyses of this intervention in patient populations, we can deduce what, if anything, is pathological about their response to this intervention. In the present study we specifically examined how social cognition training for healthy adult participants impacts recognition of and reaction times to distinct facial emotions as well as patterns of response errors. As expected, we found that, compared to participants who played control games, participants who completed social cognition training improved their recognition of distinct emotions and decreased their tendency toward making certain patterns of response errors. More specifically, social cognition training improved participants’ abilities to accurately recognize angry and neutral faces from pre- to post-intervention. It also improved how quickly they accurately identified sad, neutral, and fearful faces while marginally improving how quickly they accurately identified angry faces from pre- to post-intervention. Furthermore, social cognition training decreased participants’ tendency to mistake an angry face for no emotion (neutral) and decreased mistaking no emotion (neutral) for sadness from pre- to post-intervention. These results have implications for how to improve emotion recognition, a core social cognitive skill that supports social functioning, in both neuropsychiatric populations and healthy adults.

### Implications for individuals with neuropsychiatric disorders

Deficient recognition of angry and neutral faces has been tied to impaired social functioning, difficulty in establishing a therapeutic relationship with a clinician, and illness severity (1, 3, 17, 51–53). Therefore, this training program’s potential to improve recognition of angry and neutral faces could significantly impact the lives of individuals with Parkinson’s disease and individuals who are at risk for schizophrenia, as they both struggle with recognizing angry and neutral faces (4, 10–12, 17–21). The ability of this training program to specifically target confusing neutral faces for angry faces is also promising for individuals who have actually developed schizophrenia (18) and otherwise neuropsychiatrically healthy relatives of individuals with schizophrenia (22), both of whom tend to commit this misattribution error.

Individuals with schizophrenia (25), in addition to those with autism (54, 55), also struggle to promptly recognize facial emotions. Research has shown that faster reaction times for facial emotion recognition correlates with better social functioning (55). Therefore, the potential for this program to improve these individuals’ facial emotion recognition times may translate into improved social functioning.

Mistaking faces with no emotion (neutral) for sad faces is commonly found among individuals with major depressive disorder because of their bias toward sadness (13, 14). Interventions that target this error have been shown to improve depressive symptoms likely because they begin reversing this bias that, unchecked, perpetuates depression (15, 16). Therefore, because this social cognition training program appears to address this misattribution error, it may also have the potential to treat individuals with major depressive disorder.

Overall, this training program has the potential to bolster the social cognition of populations with various facial emotion recognition deficits.

### Implications for healthy populations

Beyond the utility of social cognition training for individuals with social cognition deficits, this study also showed that social cognition training can improve facial emotion recognition in healthy adults with above average IQ (see Table 3). Therefore, social cognition training may benefit all types of individuals and not just those with obvious deficits. To this point, our findings are in line with research showing that a contemplative emotion training program improved the abilities of neuropsychiatrically healthy school teachers to recognize facial emotions; furthermore, teachers who did not receive the training were more likely to have depressive or anxiety symptoms than those who had received the training (9).

### Mechanism of action

As explained in the introduction, we propose that this social cognition intervention improved participants’ accuracy, reaction time, and response error patterns because it utilizes the principles of neuroplasticity. Imaging data has shown that this type of training program *engages the neural networks* responsible for higher- and lower-level social cognition skills, including those responsible for facial emotion recognition (40, 41). Furthermore, because this program is *intensive, adaptive* (becomes increasingly more difficult as participants improve their performance), and *reinforcing* (i.e., rewards correct responses), we propose that it strengthens the targeted neural networks and consequently improves social cognition abilities (37).

### Future directions

Although this social cognition training program was designed to induce neuroplasticity, we did not test neural change directly. Thus, more research is necessary to examine neuroplastic response and fully understand the mechanisms supporting training-related behavioral improvements.

Furthermore, a better understanding of the mechanisms that produce training benefits will facilitate the translation of this treatment to neuropsychiatric populations that have emotion recognition deficits. Clearly, an important next step for this line of research is to directly test the effects of social cognition training in individuals with neuropsychiatric disorders, such as psychosis spectrum disorders, mood disorders, and neurocognitive disorders. Although individuals with neuropsychiatric disorders may be less neuroplastic than healthy individuals, existing data suggest that social cognition training will improve their ability to recognize distinct facial emotions given this intervention has been shown to promote neuroplastic changes (56) and improves other facets of social cognition in individuals with schizophrenia (35, 38, 42, 43).

Another important research avenue is to examine the duration of benefits from social cognition training. This can be accomplished by including assessments at longer follow-up periods to evaluate whether participants can sustain their improvements without continued training or if continued maintenance training is necessary. It is also possible that, even after training is completed, individuals will continue to improve over time as they leverage their improved social cognition abilities in real life social situations, which in turn could solidify and possibly enhance their gains.

Given the role facial emotion recognition plays in deciding how to navigate social interactions, it would be informative to investigate whether social cognition training can improve decision-making that hinges on facial emotion recognition. An appropriate task for this type of investigation would be the Go/No-go tasks used by Mancini et al. and Mirabella et al., in which healthy participants have to decide whether to proceed with or inhibit an action based on a displayed facial emotion. These researchers found being presented with an angry or fearful face, compared to other emotions, increased reaction times toward proceeding with an action but also promoted more accurate inhibition. Crucially these effects occurred just when emotional stimuli were task-relevant. By contrast, when the same images were task irrelevant (subjects were required to discriminate the actors/actresses’ gender and not their facial emotions), facial emotions did not yield any behavioral effect (57–60). It will be interesting to evaluate whether social cognition training will alter this pattern of results or perhaps make this pattern of results even more pronounced. Ultimately, future research will also need to investigate whether improvements in task performance generalizes to real world improvements in social functioning in both healthy individuals and individuals with neuropsychiatric disorders.

Finally, it will also be valuable to investigate whether social cognition training can have effects beyond improving social cognition. Given that facial emotion recognition deficits often predate the development of psychosis (4), future research should look at whether social cognition training can possibly attenuate the transition to a psychotic break Additionally, because, as stated above, our current study showed that social cognition training impacts a misattribution error that perpetuates depression (15, 16), it should be explored as an adjunctive treatment for depression.

### Limitations

This study had several limitations. Given all participants were already scoring at approximately 80% or higher for all emotions except anger (Table 5), there was little room left for improvement. This potential ceiling effect may be a reason that we did not find more improvements in accuracy within the SCT group and the reason that we did not find significant post-intervention accuracy differences between the SCT group and the CON group. Future research would benefit from using tasks that are more sensitive to training effects than the current study.

This study’s small sample size also likely masked potential training effects. For example the SCT group had numerically (although not significantly) slower reactions times to sad faces than the CON group pre-intervention; therefore, the SCT’s group significant within-subjects improvement in reaction time to sad faces only resulted in post-intervention reaction times that were comparable to those in the CON group. Larger sample sizes would likely have resulted in more comparable reaction times at baseline that would have been less likely to obscure intervention effects.

Lastly, this study did not take into account the participants’ levels of arousal for each trial. Arousal is known to impact facial emotional recognition accuracy and reaction times (61). However, facial emotion intensity has been shown to predict arousal (61, 62), and, given that emotional intensity is balanced in the Penn ER-40 task, the participants likely had comparable levels of arousal across study conditions. Nevertheless, future research should take into account participants’ arousal.

## Conclusion

Social cognition training improves recognition of angry, sad, fearful, and neutral faces. These findings are promising given there are currently limited intervention options for improving facial emotion recognition, which would benefit both individuals with neuropsychiatric disorders and even those without neuropsychiatric disorders. Future studies should explore whether social cognition training has the potential to improve impaired social functioning and poor psychological health, both of which are known consequences of impaired facial emotion recognition.

## Data availability statement

The datasets for this study are available upon request.

## Ethics statement

This study was approved by the Harvard Institutional Review Board (IRB Protocol # 14-3110). All participants received a description of the study before they provided written informed consent.

## Author contributions

CH, MN, EG, and DD-F contributed to the study’s conceptualization, methodology, investigation, and project administration. CH and MN contributed to the development of the social cognitive training program. SS, KH, AS, and CH contributed to the data curation and formal analysis. SS drafted the manuscript. All authors provided critical feedback that helped shape the research, analysis, and manuscript.
